# Detrimental role of sphingosine kinase 1 in kidney damage in DOCA-salt hypertensive model: evidence from knockout mice

**DOI:** 10.1186/s12882-020-01815-8

**Published:** 2020-05-11

**Authors:** Bingqing Lyu, Weili Wang, Xin-Ying Ji, Joseph K. Ritter, Ningjun Li

**Affiliations:** 1 School of Basic Medical Sciences, Guizhou University of Traditional Chinese Medicine, Guiyang, 550025 Guizhou China; 2 Department of Pharmacology & Toxicology, Virginia Commonwealth University School of Medicine, P.O. Box 980613, Richmond, VA 23298 USA; 3 Lingnan Medical Research Center, Guangzhou University of Chinese Medicine, Guangzhou, 510405 Guangdong China; 4 Henan International Joint Laboratory for Nuclear Protein Regulation, School of Basic Medical Sciences, Henan University College of Medicine, Kaifeng, 475004 Henan China

**Keywords:** Sphingosine-1-phosphate, α-Smooth muscle actin, Collagen, hypertension

## Abstract

**Background:**

Sphingosine-1-phosphate (S1P) is a bioactive metabolite of sphingolipids and produced by sphingosine kinases (SphK1 and SphK2). SphK1/S1P pathway is implicated in the progression of chronic kidney disease. However, the role of SphK1/S1P pathway in renal injury in hypertension has not been reported. This study tested the hypothesis that SphK1/S1P pathway mediates the kidney damage in DOCA-salt hypertensive mice.

**Methods:**

Male wild type (WT) C57BL6 and SphK1 knockout (KO) mice were subjected to unilateral nephrectomy, subcutaneous implant containing 50 mg of deoxycorticosterone acetate (DOCA) and 1% NaCl drinking water for 7 weeks. At the end of experiments, blood pressure data, 24 h urine and kidney samples were collected. Renal mRNA levels of SphK1 were measured by real-time RT-PCR. Markers for fibrogenesis and immune cell infiltration in kidneys were detected using Western blot and immunohistochemistray analysis, respectively. The glomerular morphological changes were examined in kidney tissue slides stained with Periodic-Acid Schiff. Four groups were studied: wild type control (WT-C), WT-DOCA, KO-C and KO-DOCA.

**Results:**

The renal SphK1 mRNA expression was significantly upregulated in WT-DOCA mice, whereas this upregulation of renal SphK1 mRNA was blocked in KO-DOCA mice. There was no difference in DOCA-salt-induced hypertension between WT and KO mice. The urinary albumin was increased in both DOCA-salt groups. However, the albuminuria was significantly lower in KO-DOCA than in WT-DOCA group. There were increases in glomerulosclerosis indices in both DOCA-salt groups, whereas the increases were also significantly lower in KO-DOCA than in WT-DOCA mice. Renal protein levels of α-smooth muscle actin were upregulated in both DOCA-salt groups, but the increase was significant lower in KO-DOCA than in WT-DOCA group. The increased staining areas of collagen detected by Sirius Red-staining in kidney tissue sections were also attenuated in KO-DOCA compared with WT-DOCA mice. In contrast, the increased infiltration of CD43+ (a T cell marker) or CD68+ (a macrophage marker) cells in DOCA-salt kidneys showed no significant difference between WT-DOCA and KO-DOCA mice.

**Conclusions:**

SphK1/S1P signaling pathway mediates kidney damage in DOCA-salt hypertensive mice independent of blood pressure and immune modulation.

## Background

Sphingosine-1-phosphate (S1P) plays important roles in many physiological or pathophysiological processes via its receptors on cell membrane or as an intracellular signaling molecule [1–4]. The S1P is produced by phosphorylation of sphingosine to S1P through sphingosine kinases (SphKs) [1–3]. S1P produced by SphK2 functions as an intracellular signaling molecule to generate receptor-independent effects [3, 4]. S1P produced by SphK1 acts as an extracellular signaling molecule binding to its receptors on cell membrane [4–8]. Currently, the information about SphK2-generated S1P is limited and that most of known functions of S1P are through its receptors. Five G-protein coupled receptors, S1PR1 to S1PR5 have been identified [9]. S1PR1 to S1PR3 are present in about all tissues, whereas S1PR4 and S1PR5 are mainly present in brain and lymphoid, respectively [10]. Evidence suggests that S1P is involved in different kidney diseases, such as acute kidney injury, glomerulonephritis, diabetic nephropathy, as well as renal fibrosis [11–14].

One of the significant effects by S1P is its role in fibrosis as evidence shows interaction between S1P signaling and TGF-β1 signaling to form a vicious cycle in fibrosis [15]. Fibrotic genesis is the crucial pathological process in chronic kidney diseases. Thus, S1P signaling may be involved in the renal injury in chronic kidney diseases. Indeed, it has been shown that S1PR2 and S1PR3 mediate fibrogenetic effects by interacting with Smad complexes. In addition, SphK1 level can be increased by TGF-β1 activation of Smad complex to increase S1P production. The increased S1P production may amplify fibrogenetic effects [1, 15, 16]. Although S1P signaling has been shown to participate in the pathogenesis of various kidney diseases, there is lack of evidence demonstrating the role of endogenous SphK1 in kidney damage in a hypertensive model in vivo. The present study examined the role of endogenous SphK1 in the kidney damage in deoxycorticosterone acetate (DOCA)-salt-treated mice, a hypertensive model with kidney damage, using SphK1 knockout (KO) mice.

## Methods

### Mice and DOCA-salt model

Breeding pairs of wild type (WT) and SphK1 KO mice generated on the C57BL/6 background were purchased from Jackson Laboratory. Mice were bred in our animal facility, housed in polycarbonate cages with corn cob bedding, maintained on a 12-h light/dark cycle, and fed a normal rodent chow diet with water freely available. Three month old male C57BL/6 wildtype and KO mice (25-30 g) were used. The DOCA-salt model was produced by unilateral nephrectomy and implantation of subcutaneous silicone strip (silastic, Dow Corning Co.) impregnated with 50 mg of DOCA [17, 18] when animals were anesthetized with 3% isoflurane and then fed with 1% NaCl drinking water for 7 weeks. The mean arterial pressure was monitored using a DSI telemetry blood pressure system from week 6–7. At the end of the 7th week, 24 h urines were collected for urinary albumin measurement using an ELISA kit, and kidney tissues harvested. Kidneys were cut longitudinally, half was put in 10% neutral buffered formalin for tissue sectioning, the other half in liquid N_2_ and saved in a − 80 °C freezer for molecular analysis later. Mice with sham surgery were used as controls. Mice were randomized into four groups: wild type control (WT-C), wild type DOCA-salt (WT-DOCA), SphK1 KO control (KO-C) and SphK1 KO DOCA-salt (KO-DOCA). After sample collection, mice were euthanized by exsanguinations when the animals were still under deep anesthesia with 3% isoflurane. All animal procedures were approved by the Virginia Commonwealth University’s Institutional Animal Care and Use Committee.

### Extraction of RNA and semi-quantitative analysis of mRNA levels by real time RT-PCR in kidney tissues

The extraction of total RNA was performed using TRIzol reagent purchased from Life Technology (Rockville, MD, USA). The RNA was reverse-transcribed into cDNA by a cDNA synthesis kit from Bio-Rad (Hercules, CA, USA). A TaqMan Gene Expression Assay kit (Applied Biosystems) was used for real time RT-PCR analysis using the above cDNA products. The level of 18S rRNA was measure at the same assay for endogenous control. The ΔΔCt method was used to calculate mRNA levels of SphK1. The relative mRNA levels were expressed as the values of 2^–ΔΔCt^.

### Isolation of proteins for the Western blot analysis in kidney tissues

The renal tissues were homogenized and centrifuged, and the resulting protein then used for Western blot analysis as described previously [19, 20]. Briefly, kidney samples were homogenized in ice-cold RIPA lysis buffer by using glass homogenators. The homogenates were centrifuged for 5 min at 5000 g using a refrigerated centrifuge at 4 °C. After centrifugation, the supernatant was collected and measured for protein concentration using Bio-Rad Protein Assay. The equal amount of proteins was then used for Western blot analysis. The primary antibodies used were rabbit against mouse α-smooth muscle actin (α-SMA) in 1:2000 dilution (Abcam) and GAPDH in 1:3000 dilation (Cell Signaling Technology).

### Immunostaining

The kidney samples saved in 10% neutral buffered formalin were processed into 5-μm sections after paraffin-embedment. The tissue sections were immunostained as we described previously [21, 22]. The primary antibodies were goat against mouse CD43 and rabbit against mouse CD68 (Santa Cruz Biotechnology), respectively, diluted in 1:100. The Picro Sirius Red was used for Collagen I/III staining. A computer program Image-Pro Plus was used to calculate the positive area by Picro Sirius Red staining and the number of positive cells by CD43 or CD68 staining [23].

### Evaluation of histological damages

The kidney sections were stained with Periodic-Acid Schiff (PAS) (Sigma-Aldrich staining kit) and evaluated for morphological damages. Glomerular damages were defined as collapse of capillary with increased deposition of extracellular matrix. The damages were semiquantitatively scored by two blinded examiners as described before [24, 25]. The damage scores were determined on a scale of 0–4 based on the sclerotic extent. The minimum of 20 cortical fields was examined at × 400 magnification. The scale of 0 to 4 was defined as follows: 0 = no injury; 1 = 1–25% injured area; 2 = 26–50% injured area; 3 = 51–75% injured area; 4 > 75% injured area [24, 25].

### Statistical analyses

The data were presented using mean ± standard error of the mean (SEM). The effects of gene deletion and DOCA-salt treatment were examined as independent factors by the two-way analysis of variance (ANOVA) to compare the differences among groups followed by Tukey’s test. The statistical significance was defined as a *P* value < 0.05.

## Results

### Enhanced expression of SphK1 mRNA in kidneys in DOCA-salt model

**Fig. 1:**
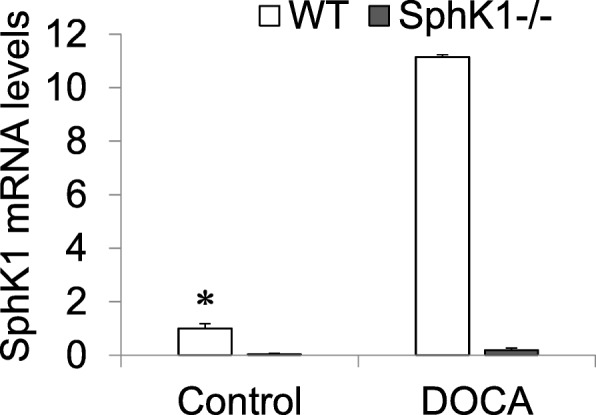
Effect of DOCA-salt and SphK1 KO on mRNA levels of SphK1 in the kidneys: Relative mRNA levels of SphK1 in different groups measured by Real time RT-PCR analysis. The results were presented as fold change normalized with the WT-C. * *P* < 0.05 vs. all other groups (*n* = 5)

The relative mRNA levels of renal SphK1 was significantly elevated by 10 fold in WT-DOCA compared with WT-C mice, whereas the mRNA levels of SphK1 mRNA was nearly undetectable in either KO-C or KO-DOCA kidneys (Fig. 1). These data demonstrated that the DOCA-salt treatment induced the expression of SphK1 in the kidneys, which was prevented in SphK1 KO mice. It is suggested that there is activation in renal SphK1 pathways in response to DOCA-salt insult.

### Attenuated albuminuria in SphK1 KO mice without effect on the hypertension in the DOCA-salt model

**Fig. 2:**
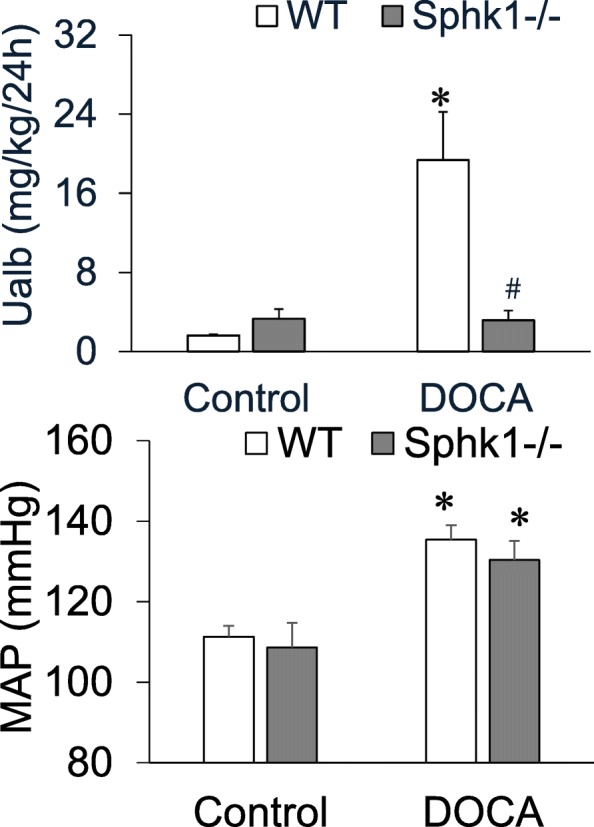
Effect of DOCA-salt and SphK1 KO on urinary albumin and blood pressure. **Upper:** Urinary albumin levels. **Lower:** Mean arterial pressure (MAP). *p < 0.05 vs. Controls; # p <0.05 vs. WT-DOCA (n = 5)

The 24 h urinary albumin excretion, an indicator of glomerular injury, was significantly increased in WT-DOCA mice compared with WT-C mice, whereas the increased urinary albumin was inhibited in KO-DOCA mice (Fig. 2). The changes in urinary albumin/creatinine ratio, a similar indicator of renal injury as the 24 h urinary albumin with correction of potential error in 24 h urine collection, showed very similar pattern among different groups (data not shown). These results demonstrated that SphK1 gene deletion attenuated DOCA-salt-induced kidney damage. This protection by SphK1 gene KO appeared independent of hypertension, because the there was no difference in blood pressure between WT-DOCA and KO-DOCA mice (Fig. 2).

### Attenuated glomerular morphological damage in SphK1 KO mice in the DOCA-salt model

**Fig. 3:**
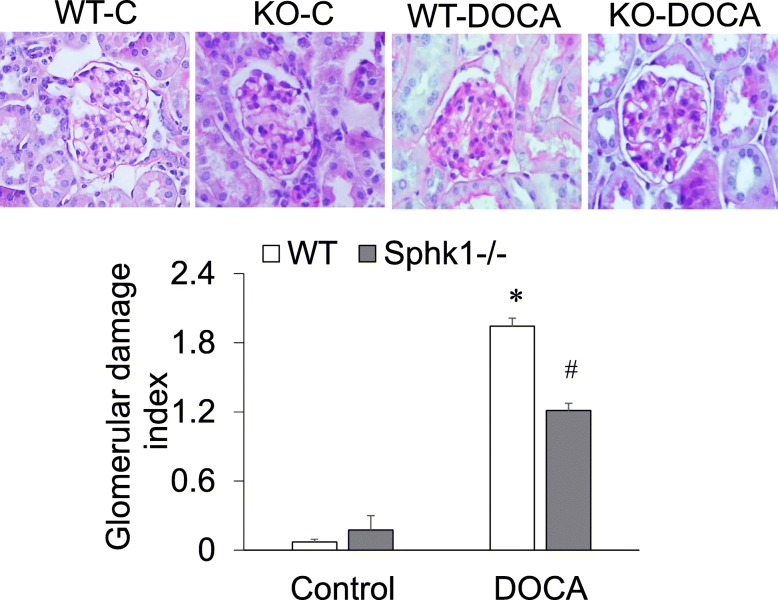
Effect of DOCA-salt and SphK1 KO on glomerular morphology. **Upper:** Representative photomicrographs of PAS staining in renal tissue slides. **Lower:** Summarized glomerular damage index by semiquantitation. * *p* < 0.05 vs. all other groups; # *p* < 0.05 vs. controls (*n* = 5)

The PAS staining in renal tissue slides showed expanded mesangial matrix with hypercellularity, capillary collapse, and fibrous deposition in the glomeruli in DOCA-salt-treated mice compared with control mice. However, these changes in glomerular morphology, as shown by the injurious scores, were notably lower in KO-DOCA than WT-DOCA mice (Fig. 3). These results indicated that the renal pathological injuries by DOCA-salt were attenuated by SphK1 deletion.

### Attenuated expression of fibrotic markers α-SMA and collagen in the kidneys in SphK1 KO mice in DOCA-salt model

**Fig. 4:**
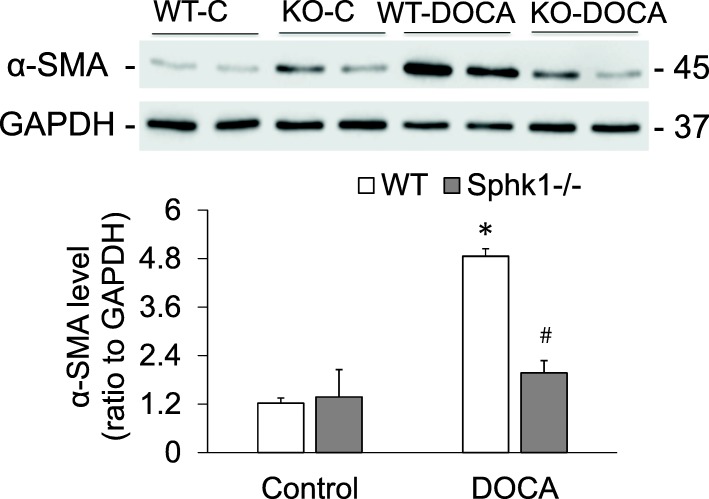
Effects of DOCA-salt and SphK1 KO on the expression of fibrotic marker α-SMA in kidney tissue by Western blot analysis. **Upper:** Representative gel documents showing α-SMA levels; **Lower:** summarized blot intensities. * p < 0.05 vs. Control; # p < 0.05 vs. WT-DOCA (n = 5)

**Fig. 5:**
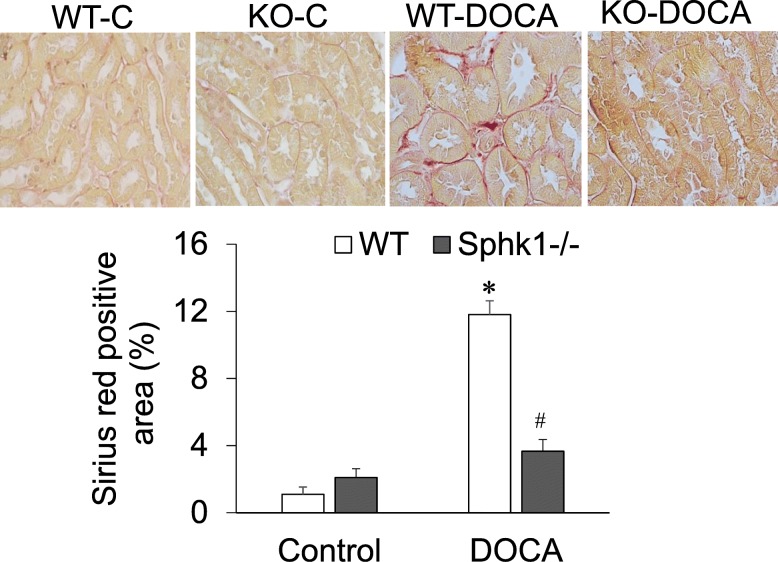
Effect of DOCA-salt and SphK1 KO on collagen staining in the kidneys. **Upper:** representative photomicrographs of Sirius Red Staining of collagen in renal tissue slides. **Lower:** quantification of the percentage of positive staining area. * *p < 0.05* vs. Control; # *p < 0.05* vs. WT-DOCA (*n* = 5)

The levels of renal α-SMA determined by Western blot analysis were significantly increased in WT-DOCA group. However, the levels of renal α-SMA were significantly lower in KO-DOCA than in WT-DOCA group (Fig. 4). The Sirius Red-positive staining area of collagen, a different fibrotic marker, was also significantly higher in kidney tissue sections in WT-DOCA groups than in control groups, but was much lower in KO-DOCA than in WT-DOCA group (Fig. 5). It is suggested that the KO of SphK1 gene inhibits fibrotic injury in the kidneys of DOCA-salt model.

### No significant difference in immune cell infiltration in kidneys between WT-DOCA and KO-DOCA

**Fig. 6:**
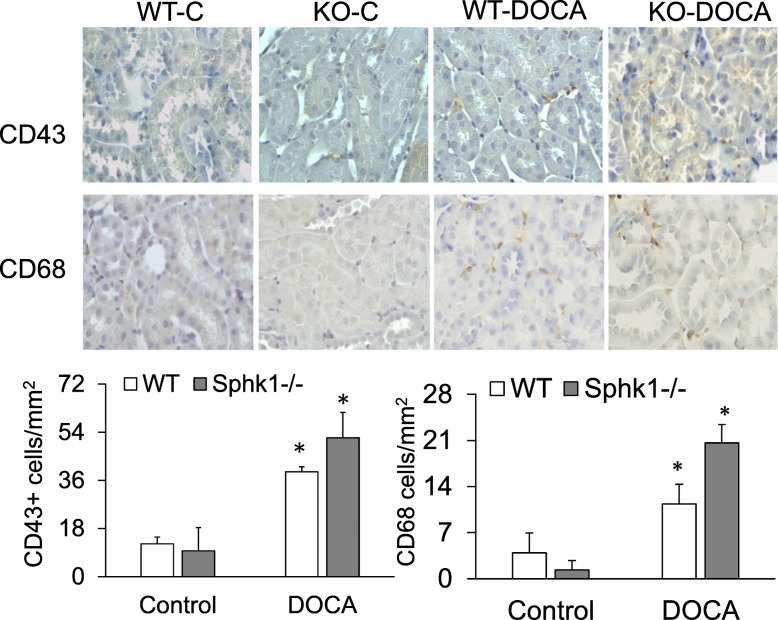
Effect of DOCA-salt and SphK1 KO on the infiltration of immune cells in the kidneys. **Upper:** Representative photomicrographs of immunohistochemistry of kidney sections stained for either CD43+ or CD68+ indicated by brown color. **Lower:** The counts of CD43+ or CD68+ cells. * *p*<0.05 vs. controls (*n* = 5)

As S1P signaling participates in immune regulation, we determined if the attenuation of kidney damages in SphK1 KO mice was produced by the changes of immune regulation due to SphK1 gene deletion and measured the renal infiltration of immune cells using immunostaining of a T-cell marker CD43 or a macrophage marker CD68. The results showed significant increases in CD43+ or CD68+ cells in both DOCA-salt groups compared with controls (Fig. 6). However, no significant difference was observed in the numbers of CD43+ or CD68 + cells between WT-DOCA and KO-DOCA groups (Fig. 6), indicating that the infiltration of immune cells was similar between WT and KO mice in DOCA-salt-treated kidneys.

## Discussion

The present study showed that renal SphK1 mRNA levels were significantly upregulated in the DOCA-salt model, that DOCA-salt-induced increases in urinary albumin, renal morphological injury scores as well as fibrotic markers, a-SMA and collagen, were significantly blocked in SphK1 KO mice, and that there was no difference in the elevation of blood pressure and increases in renal CD43+ and CD68+ cells between WT-DOCA and KO-DOCA mice. These results suggest that the SphK1 gene knockout protects kidneys against renal injuries independent of the regulatory action on immune function and hypertension in the DOCA-salt model.

We first observed a dramatic increase in the SphK1 mRNA expression in WT-DOCA kidneys compared with WT-C kidneys, which suggested the activation in S1P pathway after DOCA-salt insult. The increase of SphK1 expression in this kidney damage model was consistent with a previous finding that overexpression of SphK1 in transgenic mice produced heart fibrosis [26]. The increased SphK1 expression in the present study indicated that upregulated expression of SphK1 might participate in the kidney damage in DOCA-salt model. We thus examined if knockout of SphK1 gene to block the SphK1 increase would protect kidneys in this model.

It is worth noting that there may be a concern that a lifetime deletion of SphK1 would induce the compensation of SphK2, thereby maintaining a normal S1P level. The evidence from literature has shown that although the total S1P levels in most tissues from the SphK1 KO mice are not markedly decreased, however, in mice with a deletion of SphK1, but not SphK2, levels of S1P in interstitial fluid are greatly attenuated [27, 28]. These studies suggest that SphK2 does not compensate the reduced extracellular S1P level by SphK1 KO, probably because these two enzymes function distinctly, one for extracellular S1P production and the other for intracellular [3, 4]. The S1P produced by SphK1 acts as an extracellular signaling molecule binding to its receptors on cell membrane [4–8]. Therefore, using SphK1 KO mice would be a suitable tool to investigate the role SphK1/S1P in various diseases.

The present study showed that the albuminuria, kidney morphological changes, and increases in fibrotic markers induced by DOCA-salt were considerably attenuated in SphK1 KO mice, suggesting that overproduced S1P may be the injurious factor to produce kidney damage in this model. These results are consistent with the other reports showing that S1P participates in damages in different organ systems [29]. In line with results from the present study, exogenous S1P has been shown to induce damage in renal cell in vitro [30]. The latter study also showed that nonselective antagonist of S1P receptor or inhibitor of sphingosine kinases attenuated the renal injury and cell migration in a different kidney damage model in vivo [30]. The present study determined the role of endogenous SphK1 using a genetic knockout model in hypertensive kidney damage. Our findings suggested that SphK1/S1P/S1PRs mediates kidney damage in DOCA-salt hypertensive model.

It has been demonstrated that hypertension-induced kidney damage is largely dependent on renal perfusion pressure (RPP) in different hypertensive models [31–33] including DACA-salt model [34]. The findings from our current study demonstrated that SphK1 deletion protected the kidney without altering DOCA-salt hypertension, suggesting that SphK1/S1P pathway may also participate in RPP-induced kidney damage. In this regard, S1P pathway has been shown to participate in the mechanosensation. There are reports showing that S1P pathway is activated by shear stress and stretch in different cell types including endothelial and enthesis cells [35–37]. Although there is no evidence yet in kidney cells, these above studies, in combination with our current findings, support that activation of SphK1/S1P pathway may be a novel mechanism in pressure stress-induced kidney damage by elevated RPP, which needs future investigations. In addition, it also remains to be clarified that in what extent SphK1/S1P pathway contributes to the RPP-induced damage and DOCA-salt-induced direct damage, as well as whether this novel pathway targets both glomerular and tubulointerstitial cells at the same time or sequentially from glomeruli to tubules in this DOCA-salt model, as the filtered albumin/protein has been shown to contribute to the tubulointerstitial damage secondary to the glomerular damage [38].

Notably, the indexes for kidney damages were still significantly higher in KO-DOCA mice than in normal controls, indicating that there might be a compensated S1P production by SphK2 in SphK1 KO mice. Additionally, it is also possible that other signaling pathways independent of S1P contribute to the kidney damage in this model. In this regard, our previous studies showed that over-activation of hypoxia-inducible factor-1α pathway mediated chronic kidney damages in different models [24, 39–41]. Possible interactions may exist between S1P signaling and other pathways in renal injury, which requires further clarification.

The immune response is also involved in the pathogenesis of chronic kidney damage [42]. The infiltrations of T cells and macrophages, which represent the adaptive and innate immune responses, respectively, are observed after four-hour-obstruction in the unilateral ureter obstruction (UUO) model and the infiltration of inflammatory cells continue progressively for 2 weeks [43]. S1P has been shown to regulate multiple inflammatory processes such as immune cell recruitment into injured tissue. In addition, S1P gradients regulate the trafficking of immune cells via S1PRs, including both innate and adaptive immune cells [44]. Given the important regulatory role of S1P in immune response and inflammation, we determined whether SphK1 gene deletion attenuated the inflammatory process thereby the kidney damage in DOCA-salt kidneys. The results from immunostaining did not show a significant difference in the increases of inflammatory cell infiltrations between WT-DOCA and KO-DOCA kidneys, suggesting that the SphK1/S1P pathway probably acts directly on renal cells to produce damage, rather than on immune cells to produce kidney damage indirectly. In line with our results, a study using athymic mice with T cell deficiency showed that inhibiting S1P signaling still significantly attenuated the kidney damage induced by UUO in these immune deficient mice [45]. The findings from the present study together with other reports suggest that SphK1/S1P acts on kidney cells to produce renal injuries in chronic kidney diseases bypassing its regulation in immune response.

It should be noted that a recent study, using the same DOCA-salt model, showed that S1P agonist did not worsen the kidney damage [46]. The explanation for this discrepancy could be the different selectivity in S1P receptors between S1P and the agonist FTY720 used in the above study. The FTY720 does not act on S1PR2 [47], whereas the S1PR2-mediated signaling has been shown to play a very important role in the kidney damage [15, 48]. The renal protection by SphK1 KO in the present study is probably through the inhibition of S1PR2-mediated signaling when S1P level is reduced.

## Conclusion

In summary, the present study demonstrate that knockout of SphK1 gene protects kidneys from damage in DOCA-salt hypertensive model without significant effect on the immune cell infiltration into the kidneys neither on blood pressure, indicating that the renal protection by SphK1 deletion is independent of the hypertension and immune regulation. It is suggested that the increased production of S1P is probably a novel mechanism for chronic kidney damage in this hypertensive model and manipulating SphK1/S1P pathway may be used as novel strategies in the development of therapeutic approaches for chronic kidney diseases associated with hypertension.

## Data Availability

All data generated or analyzed during this study are included in this published article.

## References

[1] Jo SK, Bajwa A, Awad AS, Lynch KR, Okusa MD (2008). Sphingosine-1-phosphate receptors: biology and therapeutic potential in kidney disease. Kidney Int.

[2] Peters SL, Alewijnse AE (2007). Sphingosine-1-phosphate signaling in the cardiovascular system. Curr Opin Pharmacol.

[3] Maceyka M, Harikumar KB, Milstien S, Spiegel S (2012). Sphingosine-1-phosphate signaling and its role in disease. Trends Cell Biol.

[4] Maceyka M, Sankala H, Hait NC, Le Stunff H, Liu H, Toman R, Collier C, Zhang M, Satin LS, Merrill AH (2005). SphK1 and SphK2, Sphingosine kinase Isoenzymes with opposing functions in Sphingolipid metabolism. J Biol Chem.

[5] Tamama K, Okajima F (2002). Sphingosine 1-phosphate signaling in atherosclerosis and vascular biology. Curr Opin Lipidol.

[6] Liu HY, Zhang ZL, Li PY, Yuan X, Zheng J, Liu JW, Bai CQ, Niu WK. Regulation of S1P receptors and sphingosine kinases expression in acute pulmonary endothelial cell injury. Peerj. 2016;4. 10.7717/peerj.2712 PMC5157198 27994962

[7] Pyne NJ, Tonelli F, Lim KG, Long JS, Edwards J, Pyne S (2012). Sphingosine 1-phosphate signalling in cancer. Biochem Soc Trans.

[8] Maceyka M, Spiegel S (2014). Sphingolipid metabolites in inflammatory disease. Nature.

[9] Nagahashi M, Yamada A, Aoyagi T, Terracina KP, Koyama Y, Wakai T, Takabe K (2014). Sphingosine-1-phosphate signaling targeted therapy suppresses obesity-related breast Cancer progression and improves survival. J Am Coll Surgeons.

[10] Hisano Y, Nishi T, Kawahara A (2012). The functional roles of S1P in immunity. J Biochem.

[11] Geoffroy K, Troncy L, Wiernsperger N, Lagarde M, El Bawab S (2005). Glomerular proliferation during early stages of diabetic nephropathy is associated with local increase of sphingosine-1-phosphate levels. FEBS Lett.

[12] Lai LW, Yong KC, Igarashi S, Lien YH (2007). A sphingosine-1-phosphate type 1 receptor agonist inhibits the early T-cell transient following renal ischemia-reperfusion injury. Kidney Int.

[13] Awad AS, Rouse MD, Khutsishvili K, Huang L, Bolton WK, Lynch KR, Okusa MD (2011). Chronic sphingosine 1-phosphate 1 receptor activation attenuates early-stage diabetic nephropathy independent of lymphocytes. Kidney Int.

[14] Park SW, Kim M, D'Agati VD, Lee HT (2011). Sphingosine kinase 1 protects against renal ischemia-reperfusion injury in mice by sphingosine-1-phosphate1 receptor activation. Kidney Int.

[15] Zhang X, Ritter JK, Li N (2018). Sphingosine-1-phosphate pathway in renal fibrosis. Am J Physiol Renal Physiol.

[16] Koch A, Pfeilschifter J, Huwiler A (2013). Sphingosine 1-phosphate in renal diseases. Cell Physiol Biochem.

[17] Sturgis LC, Cannon JG, Schreihofer DA, Brands MW (2009). The role of aldosterone in mediating the dependence of angiotensin hypertension on IL-6. Am J Physiol Regul Integr Comp Physiol.

[18] Rhaleb N-E, Pokharel S, Sharma U, Carretero OA (2011). Renal protective effects of N-acetyl-Ser-asp-Lys-pro in DOCA-salt hypertensive mice. J Hypertens.

[19] Zhu Q, Wang Z, Xia M, Li PL, Zhang F, Li N (2012). Overexpression of HIF-1alpha transgene in the renal medulla attenuated salt sensitive hypertension in Dahl S rats. Biochim Biophys Acta.

[20] Zhu Q, Liu M, Han WQ, Li PL, Wang Z, Li N (2012). Overexpression of HIF prolyl-hydoxylase-2 transgene in the renal medulla induced a salt sensitive hypertension. J Cell Mol Med.

[21] Hu J, Zhu Q, Xia M, Guo TL, Wang Z, Li P-L, Han W-Q, Yi F, Li N (2014). Transplantation of mesenchymal stem cells into the renal medulla attenuated salt-sensitive hypertension in Dahl S rat. J Mol Med.

[22] Zhu Q, Li X-X, Wang W, Hu J, Li P-L, Conley S, Li N (2016). Mesenchymal stem cell transplantation inhibited high salt-induced activation of the NLRP3 inflammasome in the renal medulla in Dahl S rats. Am J Physiol Renal Physiol.

[23] Turnberg D, Lewis M, Moss J, Xu Y, Botto M, Cook HT (2006). Complement activation contributes to both glomerular and tubulointerstitial damage in adriamycin nephropathy in mice. J Immunol.

[24] Wang Z, Zhu Q, Li PL, Dhaduk R, Zhang F, Gehr TW, Li N (2014). Silencing of hypoxia-inducible factor-1alpha gene attenuates chronic ischemic renal injury in two-kidney, one-clip rats. Am J Physiol Renal Physiol.

[25] Thallas-Bonke V, Jha JC, Gray SP, Barit D, Haller H, Schmidt HH, Coughlan MT, Cooper ME, Forbes JM, Jandeleit-Dahm KA: Nox-4 deletion reduces oxidative stress and injury by PKC-alpha-associated mechanisms in diabetic nephropathy. Physiological reports 2014, 2(11). 10.14814/phy2.12192 PMC4255803 25367693

[26] Takuwa N, Ohkura S, Takashima S, Ohtani K, Okamoto Y, Tanaka T, Hirano K, Usui S, Wang F, Du W (2010). S1P3-mediated cardiac fibrosis in sphingosine kinase 1 transgenic mice involves reactive oxygen species. Cardiovasc Res.

[27] Nagahashi M, Yamada A, Miyazaki H, Allegood JC, Tsuchida J, Aoyagi T, Huang WC, Terracina KP, Adams BJ, Rashid OM (2016). Interstitial fluid Sphingosine-1-phosphate in murine mammary gland and Cancer and human breast tissue and Cancer determined by novel methods. J Mammary Gland Biol Neoplasia.

[28] Allende ML, Sasaki T, Kawai H, Olivera A, Mi Y, van Echten-Deckert G, Hajdu R, Rosenbach M, Keohane CA, Mandala S (2004). Mice deficient in sphingosine kinase 1 are rendered lymphopenic by FTY720. J Biol Chem.

[29] Li C, Jiang X, Yang L, Liu X, Yue S, Li L (2009). Involvement of sphingosine 1-phosphate (SIP)/S1P3 signaling in cholestasis-induced liver fibrosis. Am J Pathol.

[30] Shiohira S, Yoshida T, Sugiura H, Nishida M, Nitta K, Tsuchiya K (2013). Sphingosine-1-phosphate acts as a key molecule in the direct mediation of renal fibrosis. Physiol Rep.

[31] Mori T, Polichnowski A, Glocka P, Kaldunski M, Ohsaki Y, Liang M, Cowley AW (2008). High perfusion pressure accelerates renal injury in salt-sensitive hypertension. J Am Soc Nephrol.

[32] Mori T, Cowley AW (2004). Role of pressure in angiotensin II-induced renal injury: chronic servo-control of renal perfusion pressure in rats. Hypertension.

[33] Xia M, Li PL, Li N (2008). Telemetric signal-driven servocontrol of renal perfusion pressure in acute and chronic rat experiments. Am J Physiol Regul Integr Comp Physiol.

[34] Klanke B, Cordasic N, Hartner A, Schmieder RE, Veelken R, Hilgers KF (2008). Blood pressure versus direct mineralocorticoid effects on kidney inflammation and fibrosis in DOCA-salt hypertension *. Nephrol Dialysis Transplant.

[35] Kang H, Hong Z, Zhong M, Klomp J, Bayless KJ, Mehta D, Karginov AV, Hu G, Malik AB (2019). Piezo1 mediates angiogenesis through activation of MT1-MMP signaling. Am J Physiol Cell Physiol.

[36] El Jamal A, Briolay A, Mebarek S, Le Goff B, Blanchard F, Magne D, Brizuela L, Bougault C (2019). Cytokine-induced and stretch-induced Sphingosine 1-phosphate production by Enthesis cells could favor abnormal ossification in Spondyloarthritis. J Bone Miner Res.

[37] Duran CL, Kaunas R, Bayless KJ (2018). S1P Synergizes with Wall Shear Stress and Other Angiogenic Factors to Induce Endothelial Cell Sprouting Responses. Methods Mol Biol (Clifton, NJ).

[38] Zoja C, Abbate M, Remuzzi G (2014). Progression of renal injury toward interstitial inflammation and glomerular sclerosis is dependent on abnormal protein filtration. Nephrol Dial Transplant.

[39] Hu J, Wang W, Zhang F, Li PL, Boini KM, Yi F, Li N (2017). Hypoxia inducible factor-1alpha mediates the profibrotic effect of albumin in renal tubular cells. Sci Rep.

[40] Zhu Q, Wang Z, Xia M, Li PL, Van Tassell BW, Abbate A, Dhaduk R, Li N (2011). Silencing of hypoxia-inducible factor-1alpha gene attenuated angiotensin II-induced renal injury in Sprague-Dawley rats. Hypertension.

[41] Han WQ, Zhu Q, Hu JP, Li PL, Zhang F, Li NJ (2013). Hypoxia-inducible factor prolyl-hydroxylase-2 mediates transforming growth factor beta 1-induced epithelial-mesenchymal transition in renal tubular cells. Bba-Mol Cell Res.

[42] Meng XM, Nikolic-Paterson DJ, Lan HY (2014). Inflammatory processes in renal fibrosis. Nat Rev Nephrol.

[43] Schreiner GF, Harris KP, Purkerson ML, Klahr S (1988). Immunological aspects of acute ureteral obstruction: immune cell infiltrate in the kidney. Kidney Int.

[44] Chi H (2011). Sphingosine-1-phosphate and immune regulation: trafficking and beyond. Trends Pharmacol Sci.

[45] Yoo KH, Thornhill BA, Forbes MS, Coleman CM, Marcinko ES, Liaw L, Chevalier RL (2006). Osteopontin regulates renal apoptosis and interstitial fibrosis in neonatal chronic unilateral ureteral obstruction. Kidney Int.

[46] Iwazu Y, Muto S, Ioka T, Watanabe Y, Iwazu K, Kusano E, Nagata D (2018). Multiple sclerosis drug Fingolimod induces thrombotic Microangiopathy in Deoxycorticosterone acetate/salt hypertension. Hypertension.

[47] Brinkmann V, Billich A, Baumruker T, Heining P, Schmouder R, Francis G, Aradhye S, Burtin P (2010). Fingolimod (FTY720): discovery and development of an oral drug to treat multiple sclerosis. Nat Rev Drug Discov.

[48] Sobel K, Menyhart K, Killer N, Renault B, Bauer Y, Studer R, Steiner B, Bolli MH, Nayler O, Gatfield J (2013). Sphingosine 1-phosphate (S1P) receptor agonists mediate pro-fibrotic responses in normal human lung fibroblasts via S1P2 and S1P3 receptors and Smad-independent signaling. J Biol Chem.

